# Long-Term In Vitro Maintenance of Piglet Testicular Tissue: Effects of Tissue Fragment Size, Preparation Method, and Serum Source

**DOI:** 10.3390/ani13010128

**Published:** 2022-12-29

**Authors:** Fahar Ibtisham, Tat-Chuan Cham, Mohammad Amin Fayaz, Ali Honaramooz

**Affiliations:** Department of Veterinary Biomedical Sciences, Western College of Veterinary Medicine, University of Saskatchewan, Saskatoon, SK S7N 5B4, Canada

**Keywords:** in vitro spermatogenesis, tissue culture, infertility, organ culture systems, porcine testicular tissue

## Abstract

**Simple Summary:**

One out of every 650 children is affected by cancer, and about half of survivors become permanently infertile due to the side effects of cancer treatments. Pre-treatment testicular biopsies can be collected and cryopreserved for future use to uphold biological fatherhood. Currently, the only safe approach to produce haploid germ cells is through in vitro spermatogenesis (IVS), which has only been fully achieved in mice. Pigs are a more suitable animal model for the study of testicular development and IVS, not only because of their anatomical and physiological similarities to humans but also their long prepubertal period. This study explored optimal conditions for the long-term culture of neonatal porcine testicular tissues as a first step in achieving IVS from pigs, as a model. In this study, we investigated the effects of testicular tissue size, tissue preparation method, and serum source in media on long-term culture of neonatal porcine testicular tissues. We showed that small intact testicular tissue fragments (~2 mg) cultured in knockout serum replacement could be effectively maintained in vitro for up to 4 weeks of culture. Moreover, the presence of viable germ cells after 4 weeks of culture indicates that this approach may be applicable for IVS in pigs.

**Abstract:**

Long-term culture of testicular tissue has important applications, including the preservation of fertility potential of prepubertal boys undergoing gonadotoxic cancer treatment. This study was designed to define optimal conditions for the long-term culture of neonatal porcine testicular tissue as an animal model for preadolescent individuals. Testes from 1 wk old donor piglets were used to examine the effects of tissue fragment size (~2, 4, 6, or 8 mg), preparation method (intact, semi-digested, or physically dispersed fragments), and serum source in the media (fetal bovine serum—FBS—or knockout serum replacement—KSR). Testicular fragments were examined weekly for 4 weeks for tissue integrity, seminiferous cord density and morphology, and gonocyte counts. Testicular tissue integrity was dependent on fragment size and preparation method, where the smallest size (2 mg, *p* < 0.05) and intact preparation method were advantageous (*p* < 0.05). Seminiferous cord density decreased over the culture period (*p* < 0.05). Although the relative number of gonocytes decreased over time for all sizes and methods (*p* < 0.01), smaller intact fragments (2 and 4 mg) had greater numbers of gonocytes (*p* < 0.05). Our findings suggest that intact or physically dispersed testicular fragments of the smallest size (2 mg) cultured in KSR-supplemented media could be effectively maintained in vitro for the duration of 4 weeks.

## 1. Introduction

Recent oncological advances including safer chemotherapy and radiotherapy options have greatly improved the survivorship of cancer patients [1]. Despite these advancements, most cancer treatment options still carry serious adverse side effects, including gonadotoxicity, wherein ~46% of childhood cancer survivors face therapy-induced infertility issues as adults [2]. As the survival rate increases, so does the need to find better solutions for preserving the fertility potential of cancer patients. As a result, the collection and cryopreservation of sperm prior to starting treatment has been a standard and recommended procedure for preserving the potential for biological fatherhood in adult male patients [3]. However, this option is limited to adults only, given that prepubertal testes do not contain sperm. Nonetheless, prepubertal testes contain diploid germ cells, such as gonocytes and/or spermatogonial stem cells (SSCs), from which haploid germ cells can be potentially derived. As such, the collection and cryopreservation of testicular biopsies prior to starting gonadotoxic treatments could provide a potential source for the future production of fertilization-competent haploid germ cells from prepubertal male cancer patients.

Once testicular biopsies are collected, there are various theoretical approaches through which cryopreserved testicular tissues can be used to produce mature germ cells. These include germ cell transplantation, testicular tissue grafting, and in vitro spermatogenesis (IVS) [4,5,6]. The theoretical protocol for the auto-transplantation of germ cells involves the microinjection of testicular cells (containing SSCs) isolated from the cryopreserved testicular biopsies back into the testis of the recovered cancer patient. This procedure has been successfully applied in different animal models [7,8,9,10], but is not yet considered a safe clinical option for the purpose of preserving fertility in humans. This is because germ cell transplantation carries an inherent risk of reintroducing malignant cells, which may still be present in the pre-treatment biopsies, back into the recovered patients [6]. Alternatives to auto-transplantation of SSCs include xenogeneic transplantation of SSCs into testes of recipient animals [11], xenografting of testicular tissues under the back skin of recipient mice [12], or xeno-implantation of testicular cell aggregates under the back skin of host mice [13]. Although mature germ cells have been derived using these testis cell transfer and tissue grafting techniques from various donor species [5], and these mature germ cells are unlikely to pose a risk of cancer reintroduction, there are serious ethical, safety, and legal considerations which limit the application of approaches that rely on using host animals to produce gametes for use in humans [6].

Currently, the only perceivable safe approach to producing haploid male germ cells for the stated application is through IVS. This illusive objective has been a subject of investigation for almost a century using a broad range of two-dimensional (2D), three-dimensional (3D), and organ culture models [14,15,16]. A major step in the field of IVS was taken when mouse IVS succeeded in producing haploid germ cells, resulting in the birth of mouse offspring [17]. Interestingly, the organ culture system developed for the latter study has also supported complete IVS from the testicular tissues of an infertile mutant mouse [18] and cryopreserved testicular tissues [19]. Together, these results suggest that organ culture systems have the potential to maintain the viability of germ cells and facilitate the induction of IVS.

Systematic studies on IVS for human applications require large quantities of a consistent supply of immature donor testes, which normally would have to rely on animal models. However, the short prepubertal period in rodents and limited resemblance and applicability of rodent models of IVS to humans suggest that non-rodent animal models may be more suitable for studies on human IVS [6]. As it were, pigs serve as an ideal animal model for the study of human testicular development and IVS due to their anatomical and physiological similarities to humans, ease of access to sufficient testicular tissues through routine castrations of piglets, and their extended prepubertal period (~6 months vs. a few days in mice), while avoiding the ethical and logistic issues associated with using primate models [5].

Even though the progress in IVS using mouse donor testis cells has recently also allowed us to achieve spermatogenesis in a 2D culture system [14], most studies have shown that culturing testicular tissue fragments is a more effective method for IVS than culturing isolated testicular cells [5]. This is largely because tissue fragments maintain the necessary testis microenvironment required for both testis development and spermatogenesis. This complex microenvironment is not yet possible to mimic using 2D or 3D testicular cell cultures. However, considering the naturally long prepubertal period required to achieve IVS in porcine testicular tissue explants, the first main challenge in developing a porcine model is to determine the optimal culture conditions for the long-term maintenance of testicular architecture and functional integrity of testicular tissue samples. Therefore, the focus of the present study was to overcome this challenge by optimizing several factors related to the physical attributes of porcine testicular tissue explants and the culture conditions necessary to maintain their architecture and long-term viability.

## 2. Materials and Methods

### 2.1. Experimental Design

**Figure 1:**
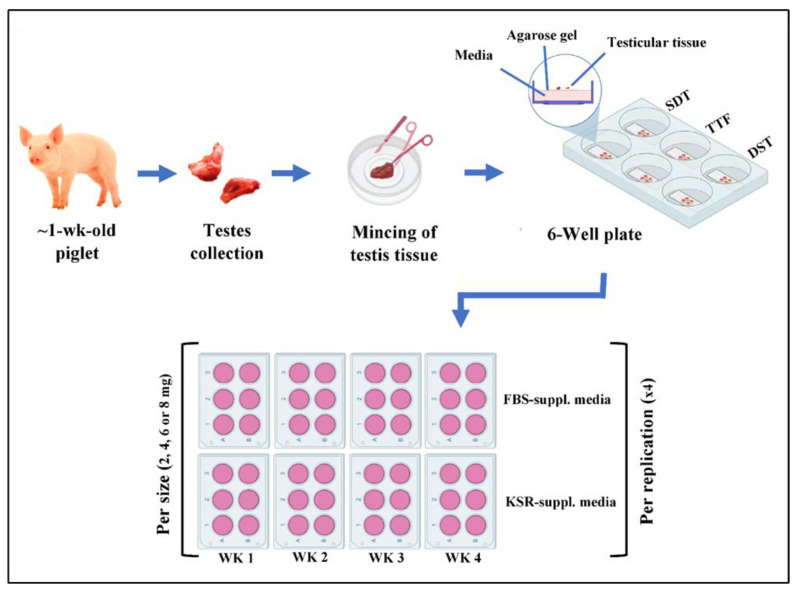
Schematic representation of the experimental design. Testes from neonatal donor piglets were divided into testicular fragments of four different sizes, underwent one of the three tissue preparation methods, and were cultured in either of the two differently supplemented media. Two fragments from each group were placed on an agarose gel cube soaked in the designated media, and samples were taken weekly for 4 weeks. PDF = physically dispersed fragments, IF = intact fragments, SDF = semi-digested fragments. FBS-suppl. = DMEM + 10% FBS culture media. KSR-suppl. = DMEM + 10% KSR culture media.

As shown in Figure 1, for the purpose of optimizing the long-term culture of piglet testicular tissue, we used a factorial design where four different sizes of testicular tissue fragments underwent one of the three preparation methods, followed by culture in either of two differently supplemented media. Two fragments from each group were placed on an agarose gel cube soaked in the designated media. Testicular tissue fragments were then cultured for 4 weeks in 6-well plates with weekly sample retrieval, where 2 wells in each plate represented a given size, preparation method, and media supplement per time point. The entire experiment was replicated four times (Figure 1). We hypothesized that (1) the long-term maintenance of testicular tissue fragments in culture and (2) the relative number of germ cells in these fragments would differ over time, depending on the fragment size, preparation method, or media supplementation.

### 2.2. Testis Collection and Preparation of Testicular Fragments

Testes from neonatal (~1 wk old) Yorkshire-cross piglets (Camborough-22 × Line 65; PIC Canada, Winnipeg, MB, Canada) were obtained from a university-affiliated swine center. Testes were collected through aseptic castration and transported in ice-cold Dulbecco’s phosphate-buffered saline (DPBS, catalogue no. 20-031-CV; Mediatech, Manassas, VA, USA) to the lab within 1 h. After rinsing 3–4 times with DPBS, tunica albuginea, rete testis, and excessive connective tissues were removed. The testis parenchyma was then cut into fragments of ~2, 4, 6, or 8 ± 0.25 mg. Each group of testicular tissue fragment sizes underwent one of the three preparation methods, including intact fragments, semi-digested fragments (3 min in 5 mg/mL of collagenase type IV, catalogue no. C-153; Sigma-Aldrich, Oakville, ON, Canada), or physically dispersed fragments using fine forceps.

### 2.3. Culture of Tissue Fragments

Testicular tissue fragments prepared using the above-mentioned methods were placed on top of 1.5% agarose gel cubes (1 × 1 × 0.5 cm; two fragments per gel cube) that were soaked in the designated media in each well of 6-well plates. Testicular tissue fragments were then cultured in an incubator under standard culture conditions (at 37 °C in 5% CO_2_) for 4 weeks in Dulbecco’s modified Eagle’s medium (DMEM, catalogue no. 10-013-CM; Mediatech), supplemented with either 10% FBS (catalogue no. A15-701; PAA laboratories, Etobicoke, ON, Canada) or 10% KSR (catalogue no. 10828028; Thermo Fisher Scientific, Carlsbad, CA, USA). The culture media were changed every 3 days.

### 2.4. Histological Analysis

Every week for 4 weeks, randomly selected samples were collected, which included all (four) fragments in a pair of designated wells per time point (Figure 1). The samples were fixed in Bouin’s solution (catalogue no. 1120-31; Richa Chemical Company, Pocomoke City, MD, USA) overnight, followed by rinsing and maintaining in 70% ethanol, processing in an automated tissue processor (Leica ASP300S, Leica Biosystems, Buffalo Grove, IL, USA), and embedding in paraffin blocks. Sections (5 µm thick) were prepared using a rotary microtome and mounted on glass slides to dry overnight on a slide warmer (37 °C). For histological assessment of cultured testicular tissue fragments, hematoxylin and eosin (H&E) staining was performed on the largest cross-sections. To analyse the integrity of basement membranes and the degree of tubulointerstitial collagen deposition, representative sections were also stained with periodic acid-methenamine silver (PAMS) and Masson’s trichrome (MT), respectively.

A semi-quantitative analysis was conducted in a blinded manner to evaluate five randomly selected fields per slide to evaluate the integrity of testis fragments. The integrity and structural changes of cultured testicular tissue sections were assessed based on the cumulative degeneration score of the basement membrane and nuclei of Sertoli cells, gonocytes, and Leydig cells (Table 1). Briefly, seminiferous cords (>30 per slide) were scored based on nucleic distinction and condensation of gonocytes and Sertoli cells, nucleoli observation of gonocytes and Sertoli cells, detachment of gonocytes and Sertoli cells from the basement membrane, disintegration of the basement membrane, and nuclei condensation of Leydig cells. The sum of the degeneration scores of nucleic changes and basement membrane morphology was calculated as the global tissue degeneration score [20]. This score ranged from 0 to −10, where 0 represented the ideal tissue integrity and −10 was designated as the worst morphology of testis fragments. In addition, the relative number of seminiferous cords per cross section was calculated (i.e., cordal density per mm^2^).

### 2.5. Immunohistochemistry

Immunohistochemistry was performed using previously reported protocols, with minor modifications [21]. Briefly, tissue sections were prepared as described above, followed by deparaffinization and dehydration. For antigen retrieval, slides were immersed in citrate buffer (pH = 6.3; catalogue no. H-3300; Vector Lab, Burlington, ON, Canada) and then in Tris-EDTA (pH = 9.3) for 40 min each at 98 °C. Samples were then rinsed with DPBS and incubated with 0.3% H_2_O_2_ for 15 min at 37 °C. The samples were then incubated with primary antibodies, UCHL1 (PGP9.5) (1:800 *v*/*v*; catalogue no. ab8189, Abcam, Toronto, ON, Canada) or GATA4 (1:200 *v*/*v*; catalogue no. sc-1237, Santa Cruz Biotechnology, Santa Cruz, CA, USA) conjugated with normal horse serum, for 1 h in a humidified atmosphere at room temperature. This was followed by rinsing with DPBS and incubation with a secondary antibody (ImmPRESS reagent peroxidase-universal anti-mouse/rabbit, catalogue no. MP7500, Vector lab) for 1 hr in a humidified atmosphere at room temperature. The sections were then rinsed with DPBS and incubated with DAB (ImmPACT DAB, catalogue no. SK4105, Vector lab) for 5–10 min, followed by counterstaining with hematoxylin for 5 min. The sections were subsequently mounted with mounting media (Sigma-Aldrich, catalogue no. 03989) and examined using light microscopy.

### 2.6. Statistical Analysis

Morphometric data of seminiferous cords were transformed and analysed using four-way analysis of variance (ANOVA) with testicular tissue fragment size, preparation method, media supplement, and week as independent factors. Seminiferous cord density was also analysed using four-way ANOVA for the same factors. The proportional (%) data of different morphology of seminiferous cords (typical or atypical) were transformed and analysed using five-way ANOVA with media supplement, testicular tissue fragment size, preparation method, week, and morphology of cords as independent factors. To compare the relative number of gonocytes per 100 Sertoli cells, four-way ANOVA was used with media supplement, fragment size, preparation method, and week as independent factors. Spearman’s rank correlation coefficient test was performed to test the relationship between the relative number of gonocytes and morphometric data.

All tests were 2-tailed, and α was set at 0.05. If a significant (*p* ≤ 0.05) interaction was found between the independent factors, data were split based on the affected factors and reanalysed using the remaining factors (e.g., three-way ANOVA instead of four-way ANOVA). Tukey’s HSD followed ANOVA tests as a post hoc to determine the pairwise differences. All analysis was performed using the Statistical Package for Social Sciences (IBM SPSS Statistics, version 25.0, IBM Inc., Armonk, NY, USA).

## 3. Results

### 3.1. Tissue Degeneration Scores of Seminiferous Cords

**Figure 2:**
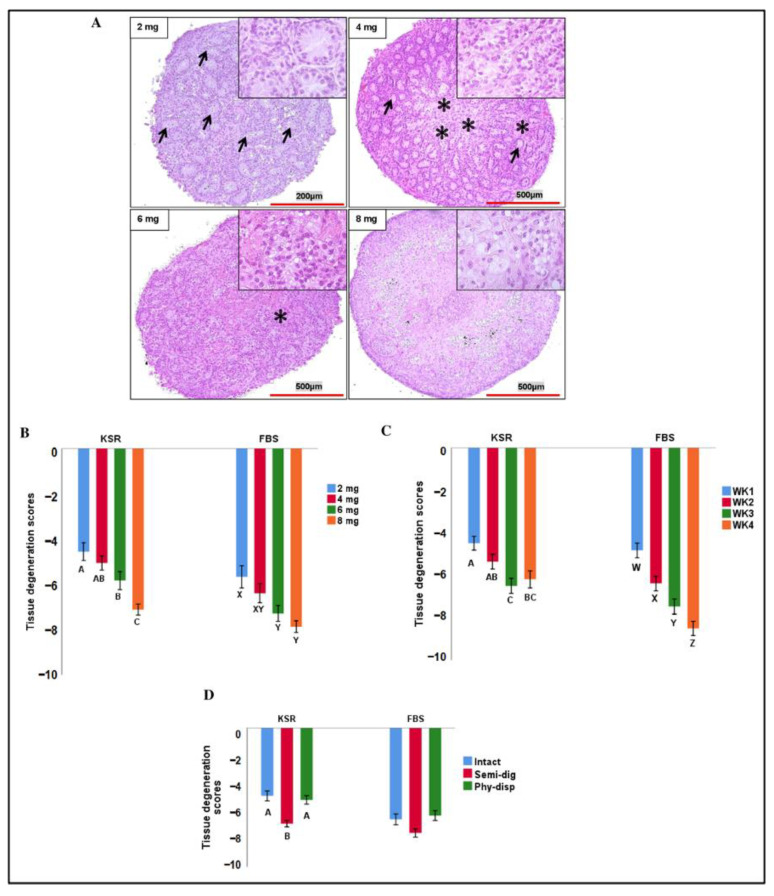
The effects of fragment size, tissue preparation method, and week of culture on tissue degeneration scores. (**A**) Representative photomicrographs of tissue fragments of different sizes retrieved at week 4 of the culture. Smallest size fragments (2 mg) better maintained their tissue integrity where seminiferous cords (arrows) were discernible throughout the tissue. Conversely, the increase in tissue fragment size that coincided with an increased loss of discernible seminiferous cords and presence of degenerated and disorganized features of spermatogenic epithelium and reduced spermatogenic cell numbers, especially in central areas, can be seen (asterisks). (**B**–**D**) A semi-quantitative histopathological assessment was conducted to compare morphological damages observed in the cultured testicular tissue fragments over time. This tissue degeneration score ranged from 0 to −10, where 0 represented no tissue damage and −10 represented most damage. (**B**) Overall, in both FBS- and KSR-supplemented media, smaller fragments had less degeneration scores than larger fragments. (**C**) In general, tissue degeneration continued over the course of culture. (**D**) In KSR-supplemented media, intact and physically dispersed (Phy-disp) fragments had less degeneration than semi-digested fragments (semi-dig); however, those in FBS-supplemented media did not differ. Data with different letters within each media supplement differ significantly (*p* < 0.05).

When comparing the global degeneration, an interaction was found between the media supplement and week of cultured testicular tissue fragments; therefore, the data were split based on media supplement (FBS and KSR) and reanalysed. For both FBS and KSR, testicular tissue fragment size, preparation method, and week of sampling all affected the degree of testicular tissue degeneration (*p* < 0.05). Comparing the effect of fragment size, overall, the smaller sizes better maintained their tissue structure (Figure 2A), where the 2 mg fragments cultured in either media supplement had less tissue degeneration compared to 6 and 8 mg sizes (*p* < 0.05, Figure 2B). Comparing the week of culture, in KSR-supplemented media, week 1 fragments had less degeneration compared to week 3 and 4, while week 2 only differed from week 3 (*p* < 0.01). However, in FBS-supplemented media, the tissue degeneration differed among all weeks, and increased over time (*p* < 0.05, Figure 2C). Comparing the tissue preparation methods showed that intact and physically dispersed tissues had less tissue degeneration compared to semi-digested tissues only in KSR-supplemented media (*p* < 0.05, Figure 2D).

### 3.2. Histopathological Findings

**Figure 3:**
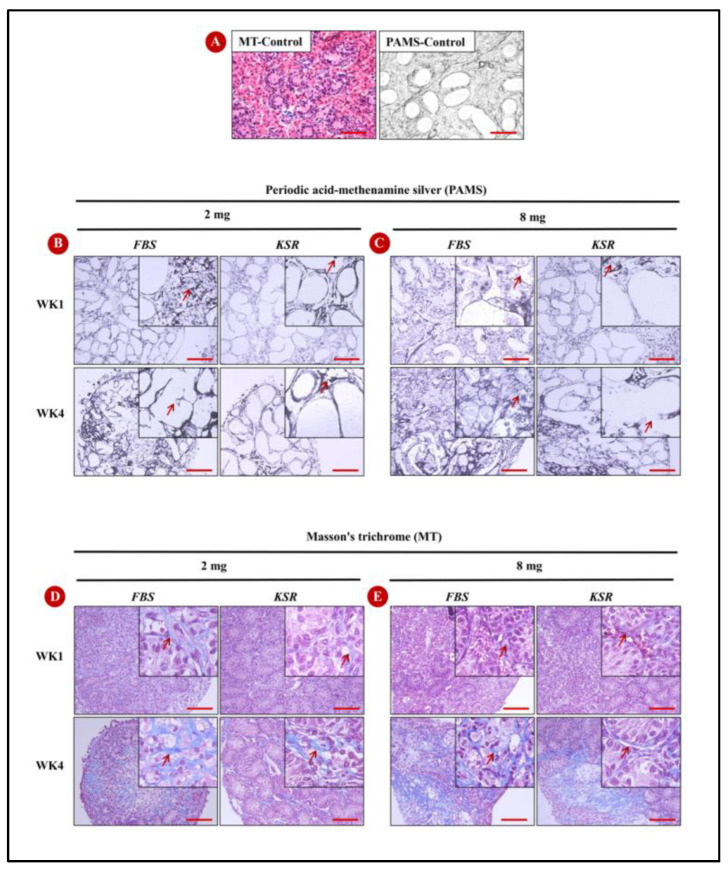
The effects of fragment size, media supplement, and week of culture on fibrosis and basement membrane of seminiferous tubules. (**A**) Representative photomicrographs of freshly isolated testicular tissue (control) stained with Masson’s trichrome (MT) to detect collagen fibers or periodic acid-methenamine silver (PAMS) to visualize the basement membrane of seminiferous cords (arrows). In these fresh samples, there are minimal collagen fibers present and no evidence of the basement membrane fragmentation. (**B**,**C**) PAMS staining of 2 and 8 mg tissue fragments, respectively, cultured in FBS or KSR for 1 or 4 weeks. Overall, tissue fragments cultured in FBS had more basement membrane fragmentation than those in KSR. (**D**,**E**) MT staining of 2 and 8 mg tissue fragments, respectively, cultured in FBS or KSR for 1 or 4 weeks. Week 4 samples of both sizes had stronger staining than week 1 samples, and tissue fragments cultured in FBS-supplemented media had stronger staining than those in KSR. Scale bar: 100 μm.

Based on the global degeneration outcomes, two sizes of cultured testicular tissue fragments, representing the best (2 mg) and worst scores (8 mg; Figure 2A) at weeks 1 and 4, were selected as representative samples to further study the histopathological changes. Testicular tissue sections were stained with MT to detect collagen fibers and PAMS to highlight the basement membrane of the seminiferous cords. In the initial control samples (1 wk old donor piglet testis), minimal collagen fibers were present, the basement membrane of seminiferous cords was intact, and no fragmentation of basement membrane was observed (Figure 3A). Staining with PAMS showed that tissues cultured in FBS had more fragmentation of the basement membrane than those in KSR. Moreover, as with the histology findings, the greater sample size had more fragmentation of the basement membrane compared to the smaller size (Figure 3B,C). Compared to week 1, week 4 samples of both sizes had excessive MT staining (Figure 3D,E), and tissues cultured in FBS-supplemented media showed stronger staining than those in KSR (Figure 3D,E).

### 3.3. Density and Morphology of Seminiferous Cords

**Figure 4:**
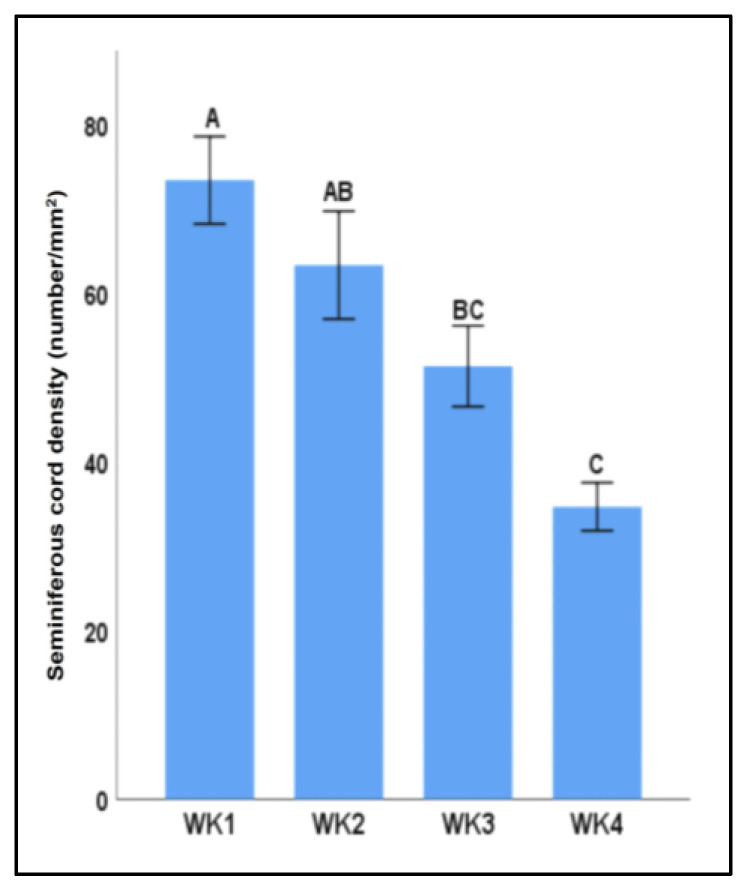
Seminiferous cord density of testicular tissue fragments over time. Density of seminiferous cords (number per mm^2^) in tissue fragments decreased over time, where week 3 and 4 samples had lower density than those in week 1 samples. Data with different letters differ significantly (*p* < 0.05).

Among all examined factors, seminiferous cord density (number of seminiferous cords per mm^2^) was only affected by the week of culture, the effect of which decreased over time (*p* < 0.05, Figure 4).

**Figure 5:**
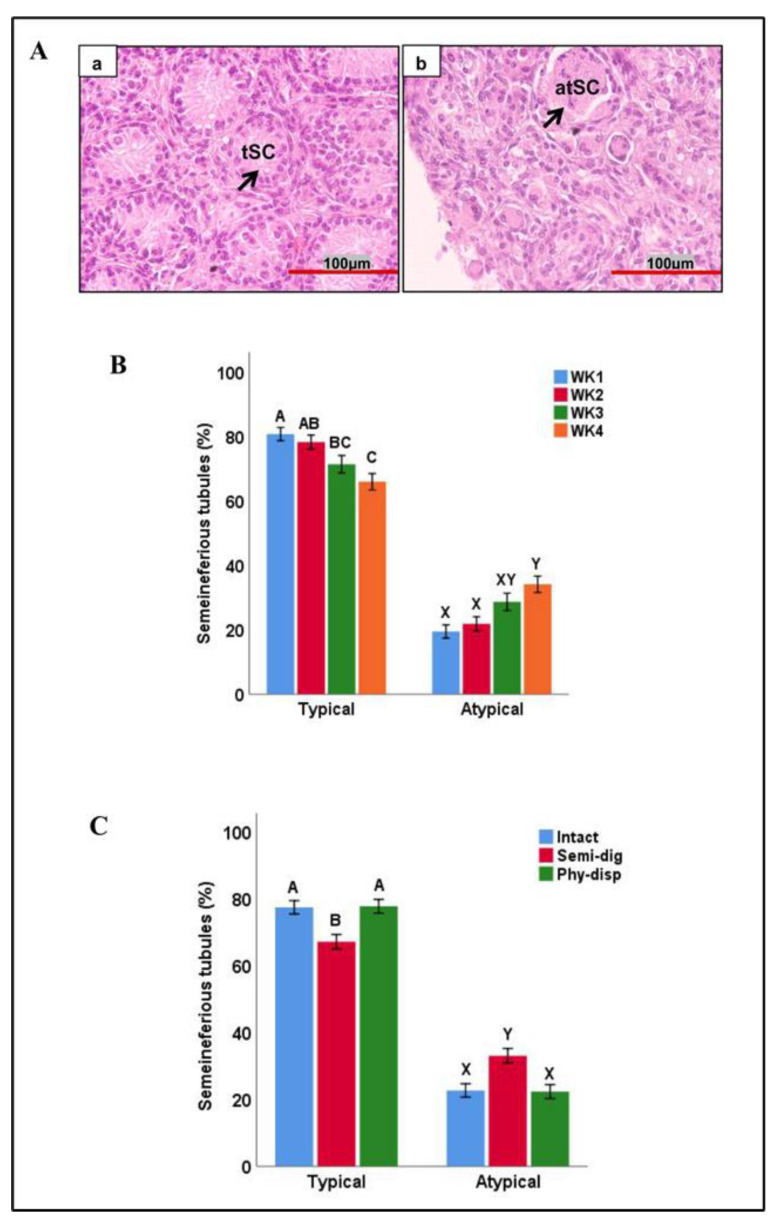
Morphology and percentage of different seminiferous cords in testicular tissue fragments during culture. (**A**) Representative photomicrographs demonstrating the morphology of seminiferous cords during in vitro culture. (**a**) Typical (tSC) seminiferous cords (arrow) in cultured tissue fragments, referring to those with a normal morphology as expected in a normal neonatal pig testis. (**b**) Atypical (atSC) seminiferous cords (arrows) in cultured tissue fragments, referring to those that did not conform to a typical morphology as expected in a normal neonatal pig testis. (**B**) The percentage of seminiferous cords displaying either morphology at different time points during culture. Overall, the parentage of typical seminiferous cords decreased, while that of atypical cords increased over time. (**C**) The percentage of seminiferous cords with either morphology in fragments undergoing each method of preparation. Intact and physically dispersed (Phy-disp) methods of fragment preparation had more typical cords and less atypical cords than semi-digested (sem-dig) method. Data with different letters within each cord morphology differ significantly (*p* < 0.05).

Upon histological examination, seminiferous cords within tissue fragments displayed various morphologies, which could be generally classified into two types: typical cords (expected in a normal neonatal pig testis) and atypical cords (those that did not conform to a typical morphology) (Figure 5A). The morphology of cords had a significant interaction with the week of culture and preparation method; hence, the data were split based on the morphology of cords and reanalysed based on week and preparation method. Based on the week of culture, week 1 had a higher percentage of typical cords than week 3 and 4 (*p* < 0.01), while week 2 had a higher percentage of typical cords than only week 4 (*p* < 0.05). Comparing the atypical cords, weeks 1 and 2 had lower percentages than week 4 (*p* < 0.01, Figure 5B). 

Morphologies of seminiferous cords also differed among the examined preparation methods. Intact and physically dispersed fragments had higher percentages of the typical type of cords and lower percentages of atypical cords compared to the semi-digested fragments (*p* < 0.01, Figure 5C).

### 3.4. Relative Number of Gonocytes

**Figure 6:**
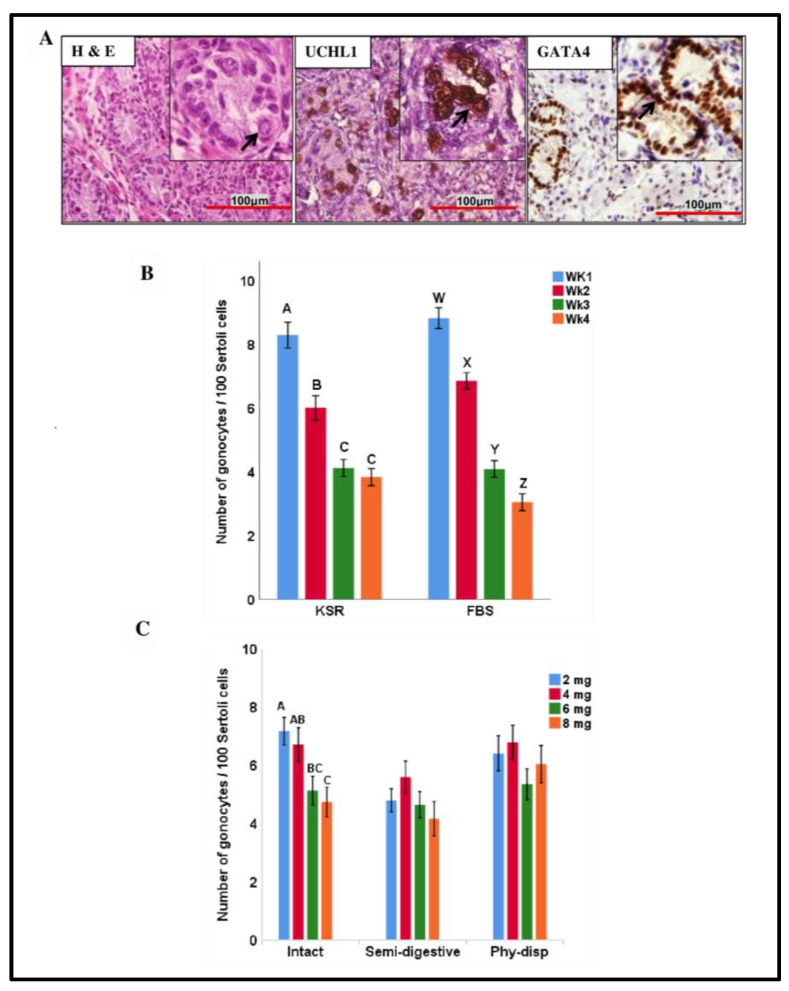
Morphology and relative number of gonocytes in testicular tissue fragments during culture. (**A**) Representative photomicrographs demonstrating the H&E staining and immunostaining of gonocytes (UCHL1) and Sertoli cells (GATA4) after 1 week of culture (arrows). (**B**,**C**) The relative number of gonocytes (per 100 Sertoli cells) was quantified in samples of tissue fragments. Overall, this ratio decreased over time for tissue fragments in both media supplementation. Among intact fragments, 2 mg tissues had more gonocytes than 6 and 8 mg, while semi-digested or physically dispersed (Phy-disp) fragments of different sizes used did not differ. Data with different letters within each media supplement or preparation method differ significantly (*p* < 0.05).

The relative number of gonocytes within samples was quantified based on the number of gonocytes per 100 Sertoli cells. The counts of gonocytes and Sertoli cells were further validated for select samples with immunostaining of UCHL1 (PGP9.5) and GATA4, respectively (Figure 6A). Since interactions were found between media supplement and week of culture as well as between preparation method and fragment size, data were split based on media supplement and preparation method. Reanalysis of data showed that for both FBS- and KSR-supplemented media, the relative number of gonocytes changed among weeks of culture (*p* < 0.01). In KSR-supplemented media, week 1 had a greater relative number of gonocytes compared to all other weeks (*p* < 0.01), and week 2 had a greater number compared to weeks 3 and 4 (*p* < 0.05). In contrast, all weeks were different from each other in FBS-supplemented media (*p* < 0.01), where weeks 1 and 4 had the highest and lowest relative number of gonocytes, respectively (Figure 6B).

Among tissue preparation methods, only in the intact group, various sizes of tissue fragments differed in their relative gonocyte numbers (*p* < 0.05), wherein the 2 mg fragments group had relatively more gonocytes compared to 6 and 8 mg groups, and the 4 mg group had more gonocytes than the 8 mg group (*p* < 0.05; Figure 6C). Comparing the tissue preparation methods, fragments in intact and physically dispersed groups contained relatively more gonocytes than the semi-digested group (Figure S1). A significant negative correlation was found between the relative number of gonocytes and tissue degeneration scores (*r* = −0.285, *p* < 0.01; Figure S2).

## 4. Discussion

This study set out to take the first steps in establishing a porcine model of long-term in vitro culture of immature testicular tissue by optimizing the physical attributes of testicular tissue samples. We investigated the effects of different testicular tissue fragment sizes, preparation methods, and media supplementation during the 4 weeks of tissue culture. Our data suggest that intact and physically dispersed fragments of ~2 mg can effectively maintain the cultured tissue integrity, cord morphology, and number of gonocytes over the period of culture.

To establish in vitro culture conditions that are more representative of the in vivo environment, we used a gas–liquid interface system (organ culture model). This system facilitates the proper diffusion of nutrients from media while allowing gas exchange to cultured tissues. We observed higher degeneration of testicular tissue with increasing tissue sizes, which could be attributed to insufficient nutrient diffusion and/or poor gas exchange to the core of larger-sized tissue fragments. These findings agree with a previous report that observed central degeneration of murine testicular tissue cultured on a half-socked agarose gel, which could be ameliorated using a microfluidic system [22]. Later, it was reported that flattening or spreading the testicular tissue on agarose gel not only prevented central necrosis but also led to increased tissue growth/volume over time [23]. These findings suggest that increasing the available surface area of cultured fragments could improve the diffusion of nutrients and gas exchange to the cultured tissues. Therefore, in the present study, we dispersed the testicular tissue using either digestive enzymes or physically using forceps. However, we observed that the dispersion of testicular tissue did not improve the outcomes. Moreover, the enzymatic dispersion of tissues resulted in significantly more tissue degeneration, perhaps due to the side effect of enzymes on the viability and activity of cells [24].

In terms of the media supplementation, FBS and KSR are the most commonly used supplements for testicular tissue cultures [17,25]. However, it was shown that for mouse testicular tissue culture, KSR is a better supplement for efficient in vitro spermatogenesis [17]. We also observed that overall, KSR-supplemented media was considerably better than FBS in maintaining the tissue architecture of testis fragments in culture. In FBS-supplemented media, testicular tissue was further degenerated after each week of culture. Moreover, we observed a high level of collagen fiber deposition and loss of integrity of the seminiferous cord basement membrane in FBS-supplemented media over time, especially when compared to that of KSR-supplemented media. Oxidative stress has been suggested to play a key role in the induction of tissue fibrosis, which is an irreversible change in many reproductive pathologies. Oxidative stress stimulates the production of reactive oxygen species (ROS), a chemical stressor which in turn is capable of activating many cell-damaging transcription factors that lead to inflammation and fibrosis of the tissue [26,27]. In the present study, increased interstitial testicular fibrosis as indicated by excessive MT staining could also be partly attributed to oxidative stress led by improper perfusion of nutrients and/or poor gas exchange to the cultured tissue. Moreover, high levels of fiber deposition in testicular tissues cultured in FBS-supplemented media may explain the heightened rate of testicular tissue degeneration observed in this group over time. However, this mechanism remains unclear, and whether such stress and/or ROS was responsible for the observed damages, especially in the FBS-supplemented tissue, remains to be investigated in future studies.

In this study, we observed that the cordal density decreased over time. Furthermore, during the culture of testicular tissue, we observed different morphologies of seminiferous cords, which we categorized as either typical or atypical. Interestingly, we observed that over the course of culture, the percentage of typical cords decreased while atypical cords increased. Overall, numerically, the smallest testicular tissue samples (2 mg) had the highest number of typical and lowest number of atypical cords. In contrast, the largest fragment size (8 mg) had the lowest number of typical and highest number of atypical cords (Figure S3). In terms of testicular tissue preparation methods, intact and physically dispersed fragments better maintained their typical cords over the period of culture, while the semi-digested method had the highest number of atypical cords. The increased number of atypical cords could also be due to the adverse effects of digestive enzymes on cellular integrity and function [24].

In studies using similar models, the main rationale for using a gas–liquid interface [17], microfluidic devices [22], or pumpless microfluidic devices [28] for testicular tissue culture was to optimally maintain the viability of both germ and somatic cells during the period of culture for achieving IVS. Gonocytes are the only type of germ cells present in the neonatal pig, so maintaining their population during in vitro culture of testicular tissue is of great interest for achieving IVS. Based on H&E staining, we did not observe germ cell development beyond the gonocyte/spermatogonia stage during the 4 weeks of culture. Therefore, for cross-validation of germ cell counts, testicular tissue fragments were labelled with UCHL1 (also known as PGP9.5), which is expressed in early germ cells (gonocytes/spermatogonia) of different species, including pigs [29], humans [30], bulls [31], and cats [32]. We observed a significant reduction in the relative number of gonocytes over the period of culture in both KSR- and FBS-supplemented media. However, this was not surprising, because in this study we did not use any additional supplements such as growth factors in our culture. Future studies may include the effects of various growth factors on maintenance of gonocytes/spermatogonia as indicated in other settings [33] for cultured testicular tissue. Moreover, we observed a negative correlation between tissue degeneration score and the relative number of gonocytes, suggesting that as expected with higher tissue degeneration, the number of gonocytes decrease. To the best of our knowledge, this is the first report of maintaining pig testicular fragments in culture for as long as 4 weeks.

## 5. Conclusions

In this study, we evaluated the effects of testicular tissue fragment size, method of tissue preparation, and culture media supplementation over 4 weeks of culture. Our findings suggest that in vitro culture of small (2 mg) intact or physically dispersed testicular fragments in KSR-supplemented media could be effectively maintained for the duration of at least 4 weeks. The presence of viable germ cells (gonocytes) after long-term in vitro culture without any growth factors indicates that this approach may be viable for IVS in pigs, although no development of germ cells was observed during the 4 weeks of culture. However, the optimal conditions to achieve complete IVS in neonatal pigs still need to be established as a suitable model to uphold the biological fatherhood of prepubertal boys undergoing gonadotoxic treatment.

## Figures and Tables

**Table 1:** A semi-quantitative scoring system for the assessment of integrity and the structural changes of cultured testicular tissue fragments.

Nuclei of Sertoli Cells and Gonocytes						Basement Membrane				Nuclei of Leydig Cells	
Distinction between Sertoli and gonocyte nuclei		Visibility of Sertoli and gonocyte nucleoli		Nuclei condensation		Detachment of cells from the basement membrane		Fragmentation of the basement membrane		Nuclei condensation	
**Score**	**Criteria**	**Score**	**Criteria**	**Score**	**Criteria**	**Score**	**Criteria**	**Score**	**Criteria**	**Score**	**Criteria**
0	Easy	0	Visible in >40%	0	Completely absent	0	Completely absent	0	Completely absent	0	In <40%
−1	Difficult	−1	Indistinguishable	−1	In <40% of nuclei	−2	Partial	−1	Obvious fragmentation	−1	In >40%
−2	Impossible			−2	In >40% of nuclei	−3	In >75% of the circumference				

## Data Availability

If additional data related to this study are required, please consult the corresponding authors.

## Supplementary Materials

The following supporting information can be downloaded at: https://www.mdpi.com/article/10.3390/ani13010128/s1, Figure S1: Tissue preparation method and relative number of gonocytes in testicular tissue fragments during culture, Figure S2: Correlation between tissue degradation scores and relative number of gonocytes, Figure S3: Morphology and percentage of different seminiferous cords in testicular tissue fragments during culture.
