# Supplementary material for: Long-Term In Vitro Maintenance of Piglet Testicular Tissue: Effects of Tissue Fragment Size, Preparation Method, and Serum Source
Source: Animals (Basel). 2022 Dec 29;13(1):128. doi: 10.3390/ani13010128 (PMC9817678; doi:10.3390/ani13010128)
Supplement: Supplementary file 1 [file animals-13-00128-s001.zip › animals-2110299-supplementary.pdf]

Supplementary Figure S1

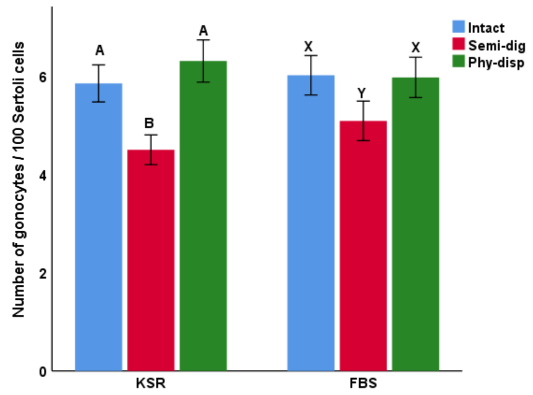

Supplementary Figure S1. Tissue preparation method and relative number of gonocytes in testicular tissue fragments during culture. Intact fragments and physically dispersed (Phy-disp) groups had significantly higher relative number of gonocytes (per 100 Sertoli cells) than the semi-digested (Semi-dig) group in both media supplementations. Data with different letters within each media supplement or preparation method differ significantly ($p < 0.05$).

Supplementary Figure S2

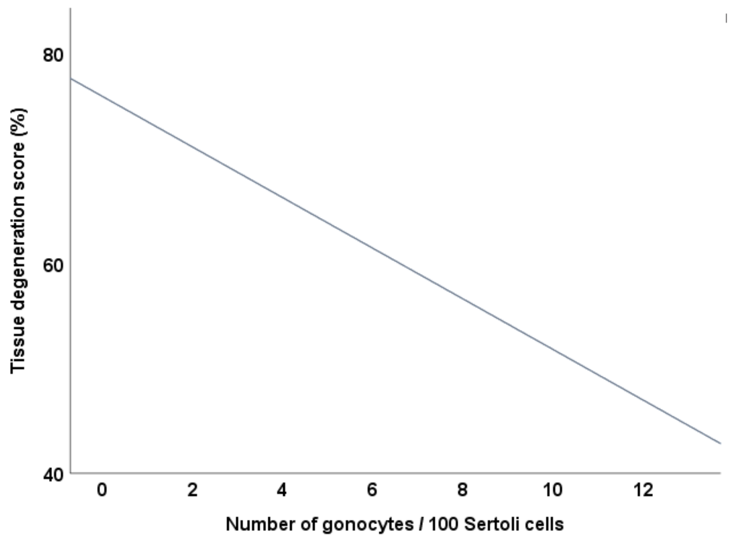

Supplementary Figure S2. Correlation between tissue degradation scores and relative number of gonocytes. A negative correlation was observed between the two factors suggesting that with higher tissue degeneration, the number of gonocytes decrease.

Supplementary Figure S3

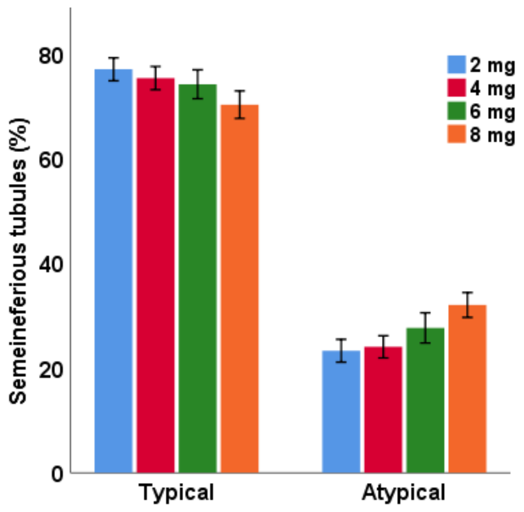

Supplementary Figure S3. Morphology and percentage of different seminiferous cords in testicular tissue fragments during culture. Although not significantly different, the smallest testicular tissue samples (2 mg) had the highest number of typical and lowest number of atypical cords. In contrast, the largest fragment size (8 mg) had the lowest number of typical and highest number of atypical cords.
